# Osteoid osteoma of the scaphoid bone mimicking juvenile idiopathic arthritis

**DOI:** 10.1186/1546-0096-10-S1-A34

**Published:** 2012-07-13

**Authors:** Gaëlle Chédeville

**Affiliations:** 1 The Montreal Children's Hospital, Montreal, QC, Canada

## Purpose

Osteoid osteoma is a benign bone tumor usually seen in long bones. Intraarticular involvement is very rare, especially in the carpal bones. In these cases, the diagnosis may be challenging as the symptoms and imaging may not be typical. Herein is the description of a boy diagnosed with an osteoid osteoma of the left scaphoid bone, who presented with a severe wrist arthritis.

## Results

A 15 yo boy was referred to Rheumatology for left wrist swelling. His past medical history was unremarkable until 8 months prior when he began to have significant pain and progressive swelling of the left wrist. He was initially diagnosed with a fracture of the scaphoid bone for which he was casted for 2 months. After the cast was removed, the patient noticed that his symptoms persisted. The pain worsened and started to wake him from sleep. After a few visits to the emergency room and another cast, he was referred to Rheumatology. The physical examination was consistent with a significant arthritis of the left wrist. The wrist was diffusely swollen, warm, very painful to touch and to movement, and the range of motion was significantly limited. No other joints were involved. An X-ray of the left wrist revealed marked joint space narrowing of the carpal bones with significant osteopenia but no other abnormalities. A diagnosis of Juvenile Idiopathic Arthritis (JIA) was made. NSAIDs were started and a joint injection was performed. No change in symptoms or physical examination was observed. The patient was taking codeine regularly for pain relief. Therefore 3 months after, a 2^nd^ joint injection was performed, with no effect. Repeat X-rays and an MRI were requested to better delineate the inflammatory process before starting methotrexate. X-rays revealed that a round sclerotic lesion had become evident in the left scaphoid bone, which was confirmed by the MRI. This was consistent with an osteoid osteoma. The MRI also revealed marked diffuse synovitis in the left wrist associated with erosive disease and joint space narrowing. The patient was referred for radiofrequency ablation. After a few months, the inflammatory process of the left wrist improved significantly with only minimal residual swelling. The patient is now off pain medication and is only taking an NSAID intermittently. Repeat X-rays demonstrate no further evidence of the osteoid osteoma but the arthritic changes of the carpal bones persist.

## Conclusion

Intraarticular osteoid osteoma can be difficult to diagnose. Secondary synovitis has been described in the hips and elbows but only one case of degenerative arthritis has been reported, which was in the carpal bones. In our patient, the non response to medical therapy and the significant degree of pain in a single joint pointed towards a diagnosis other than JIA. In such situations, one should consider the diagnosis of osteoid osteoma. Repeating X-rays and performing other imaging modalities can help uncover the right diagnosis.

## Disclosure

Gaëlle Chédeville: None.

